# BARIATRIC SURGERY AS A TREATMENT FOR IDIOPATHIC INTRACRANIAL HYPERTENSION IN A MALE ADOLESCENT: CASE REPORT

**DOI:** 10.1590/1984-0462/2020/38/2018239

**Published:** 2020-01-13

**Authors:** Marina Ybarra, Tiago Jeronimo dos Santos, Edjane Santos Queiroz, Ludmilla Rachid, Ruth Rocha Franco, Louise Cominato, Frederico Castelo Moura, Manoel Carlos Velhote, Durval Damiani

**Affiliations:** a Universidade de São Paulo, São Paulo, SP, Brazil.

**Keywords:** Pseudotumor cerebri, Obesity, Bariatric surgery, Adolescent, Pseudotumor cerebral, Obesidade, Cirurgia bariátrica, Adolescente

## Abstract

**Objective:**

To describe a case of a male adolescent with symptomatic idiopathic intracranial hypertension (IIH) associated with obesity treated with bariatric surgery.

**Case description:**

A 16-year-and-6-month-old severely obese boy [weight: 133.6 kg; height: 1.74 m (Z score: +0.14); BMI: 44.1 kg/m^2^ (Z score: +4.4)], Tanner pubertal stage 5, presented biparietal, high-intensity, and pulsatile headaches, about five times per week, associated with nocturnal awakenings, and partial improvement with common analgesics, for three months. Ophthalmologic evaluation evidenced bilateral papilledema. Cranial computed tomography revealed no mass or anatomic abnormalities. Lumbar puncture showed increased intracranial pressure of 40 cmH_2_O (reference value: <28 cmH_2_O) with a normal content. After being diagnosed with IIH, the patient was started on acetazolamide. However, after three months, he was still symptomatic. He was diagnosed with obesity due to excess energy intake and, as he had failed to lose weight after a conventional clinical treatment, bariatric surgery was indicated. The patient (at 16 years and nine months) underwent an uncomplicated laparoscopic sleeve gastrectomy. Ophthalmologic evaluation, performed five months after surgery, revealed normal visual acuity in both eyes and improvement of bilateral papilledema. Follow-up at 18 months showed a 67.5% loss of excess weight (weight: 94.5 kg and BMI: 31.2 kg/m^2^) and complete resolution of IIH symptoms.

**Comments:**

IIH is characterized by increased intracranial pressure with no evidence of deformity or obstruction of the ventricular system on neuroimaging. It has been associated with obesity. Bariatric surgery may be a valid alternative approach for morbidly obese adolescent patients with refractory symptoms.

## RESUMO

**Objetivo:**

Descrever um caso de cirurgia bariátrica como tratamento de pseudotumor cerebral primário (PTCP) em adolescente do sexo masculino com obesidade.

**Descrição do caso:**

Adolescente, sexo masculino, 16 anos e 6 meses, com obesidade exógena [peso:133,6 kg; estatura:1,74 m (escore z: +0,14); IMC: 44,1 kg/m^2^ (escore z: +4,4)], estadiamento puberal de Tanner 5, apresentando cefaleia bi-parietal, pulsátil e de alta-intensidade, cerca de cinco vezes por semana, associada a despertares noturnos, e com melhora parcial com analgésicos comuns, há três meses. A avaliação oftalmológica evidenciou papiledema bilateral e a tomografia computadorizada de crânio não revelou massas ou alterações anatômicas. A punção lombar mostrou pressão intracraniana elevada de 40 cmH_2_O (Referência: <28 cmH_2_O) com conteúdo normal. Feito o diagnóstico, o paciente foi iniciou uso de acetazolamida. No entanto, após 3 meses, o paciente mantinha-se sintomático. Ele foi diagnosticado com obesidade devido ao consumo calórico excessivo e, como não havia obtido sucesso na perda de peso com tratamento clínico convencional, a cirurgia bariátrica foi indicada. Aos 16 anos e 9 meses, o paciente foi submetido a gastrectomia vertical laparoscópica sem complicações. A avaliação oftalmológica, cinco meses após a cirurgia, revelou melhora do papiledema bilateral com acuidade visual normal em ambos os olhos. Apresentou perda de excesso de peso de 67,5% (peso: 94,5 kg e IMC:31,2 kg/m^2^) e resolução completa dos sintomas de PPTC 18 meses após a cirurgia.

**Comentários:**

O PTCP é caracterizado pelo aumento da pressão intracraniana, sem evidência de deformidade ou obstrução do sistema ventricular na neuroimagem. Está associado à obesidade. A cirurgia bariátrica pode ser uma alternativa terapêutica válida para pacientes adolescentes obesos graves com sintomas refratários.

## INTRODUCTION

Idiopathic intracranial hypertension (IIH), also known as primary pseudotumor cerebri, is clinically characterized by increased intracranial pressure in an alert and oriented patient, with no evidence of deformity or obstruction of the ventricular system on neuroimaging.^1^ Cerebrospinal fluid (CSF) analysis is normal except for an increased intracranial pressure at the lumbar puncture,^1^ greater than the 90th percentile (28 cmH_2_O) in the pediatric population.^2^ Papilledema may or may not be present.^3^

Headache is the most common symptom of IIH (84%) and is often described as daily, bilateral, frontal, or retro-ocular. Visual loss is the main morbidity of IIH, and transient visual disturbances can occur in up to 68% of patients.^4,5^

Obesity is a consistent risk factor for the development of IIH. Body mass index (BMI) has been associated with risk of IIH.^2^ IIH in adolescents appears to have similar characteristics to those in adults, including the association with obesity.^3^ Early diagnosis and treatment of IIH are imperative to prevent permanent vision loss.^5^ Our objective was to describe a case of a male adolescent with symptomatic IIH associated with obesity and treated with bariatric surgery.

## CASE DESCRIPTION

A 16-year-and-6-month-old severely obese boy [weight: 133.6 kg; height: 1.74 m (+0.14 standard deviation — SD); BMI: 44.1 kg/m^2^ (+4.4 SD)], Tanner pubertal stage 5, followed for obesity due to excess energy intake in our Pediatric Endocrinology Clinic since he was eight years old, and with a history of severe obstructive sleep apnea, gastrointestinal reflux disease, depression, insulin resistance (HOMA-IR 9.8), moderate hepatic steatosis [based on ultrasound findings and ALT: 41 U/L (reference value: <40 U/L)], and systemic arterial hypertension with cardiac left ventricular hypertrophy, presented biparietal, high-intensity, and pulsatile headaches.

**Figure 1:**
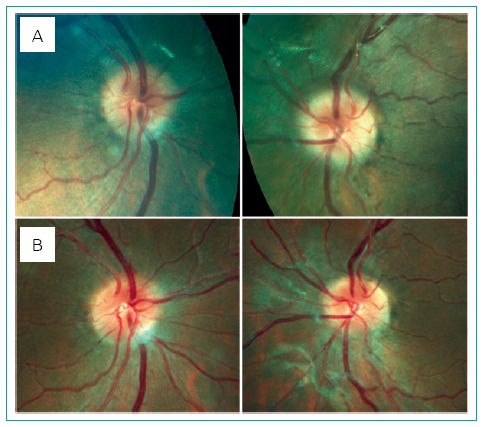
Eye fundus photography before (A) and after (B) laparoscopic sleeve gastrectomy. Note the improvement of bilateral papilledema.

The headaches had progressively worsened over the prior three months. They occurred five times per week and were associated with nocturnal awakenings. There was partial improvement with common analgesics. He was not able to stand still or walk straight without falling during the headache episodes. Ophthalmologic evaluation confirmed bilateral papilledema (Figure 1A), normal visual acuity, and absence of abducens nerve palsy. Cranial computed tomography revealed no mass or anatomic abnormalities. Lumbar puncture showed increased intracranial pressure of 40 cmH_2_O (reference value: <28 cmH_2_O) with a normal content. Optical coherence tomography (OCT) was not performed.^6^ IIH was diagnosed. The patient was started on acetazolamide q12h with partial improvement of his symptoms. However, after three months, he was still symptomatic.

As he had already failed to lose weight after being enrolled in a medically supervised weight-loss program (composed of a multidisciplinary team including a nutritionist, physical therapist, psychologist, and pediatric surgeon specialized in bariatric surgery), and exhibited a bone age of a 17-year-old, we indicated bariatric surgery. During this period, he and his family were encouraged to make lifestyle changes (healthy diet and physical activity). They were also followed monthly by a psychologist. The patient was treated with sibutramine, fluoxetine, and metformin, but showed no response. Our decision was taken after considering the criteria established by the Brazilian Federal Council of Medicine guidelines to undergo bariatric surgery in adolescence,^7^ which the patient fulfilled. The family formally consented, and the patient assented to the procedure.

At the age of 16 years and nine months, the patient underwent an uncomplicated laparoscopic sleeve gastrectomy. Ophthalmologic evaluation, performed five months after surgery, revealed normal visual acuity in both eyes and improvement of bilateral papilledema (Figure 1B). Follow-up at 18 months showed a 67.5% loss of excess weight (weight: 94.5 kg and BMI: 31.2 kg/m^2^) and complete resolution of IIH symptoms. Insulin resistance (HOMA-IR: 2.4) and hepatic steatosis normalized, and antihypertensive drugs were no longer needed.

## DISCUSSION

We present a case of a severely obese male adolescent with IIH who had complete symptom resolution with bariatric surgery after a failed clinical treatment.

Although there is no current consensus on the best management strategy for IIH, the goals should be to preserve visual function and reduce long-term headache disability.^8^ In adults with obesity-related IIH, weight reduction — either by diet or bariatric surgery — improved vision, with papilledema and IIH resolution.^9,10^ Surgical interventions were associated with 100% of postoperative IIH resolution against 66.7% in the non-surgical group (95%CI 45.6–87.8; p<0.005).^11^ Some authors even consider bariatric surgery as the procedure of choice for severely obese patients with IIH.^11^ A prospective randomized trial in adults is currently evaluating its effectiveness.^12^

There is a paucity of evidence-based recommendations for the treatment of IIH in children or adolescents.^1^ According to the International and the Brazilian Guidelines, adolescents with a BMI greater than 35 kg/m^2^, associated with severe comorbidities and complete growth plate (epiphyseal cartilage) closure, may clinically benefit from surgical weight loss.^7,13^ Chandra et al. published a case report which demonstrated complete resolution of IIH symptoms after a gastric bypass in an adolescent girl.^14^ Other reports also showed the safety of the bariatric surgery as a treatment for IIH.^15-17^ Laparoscopic sleeve gastrectomy could be an alternative surgery since it has already proven to be safe and effective in the treatment of morbidly obese adolescents.^18^ Mortality rates after bariatric surgery are low.^19^ A wide range of surgical complications may occur after bariatric surgery. Pulmonary and venous thromboembolism occur in <0.5% of bariatric surgery patients, usually within the first postoperative month.^20^ Other complications are procedure-specific and may include anastomotic leak, anastomotic stricture, bowel perforation, hemorrhage, incisional hernia, and marginal ulcer.^21^ Common gastrointestinal side effects after bariatric surgery include: vomiting, diarrhea, dumping syndrome, hypoglycemic syndrome, and cholelithiasis.^22^ Micronutrient deficiencies may also occur after bariatric surgery.^22^

Our case shows that bariatric surgery may be a valid alternative approach for morbidly obese adolescent patients with refractory symptoms. Our patient presented complete resolution of IIH signs and symptoms and experienced a 67.5% loss of excess weight after surgery.
